# Dilated perivascular spaces and steno-occlusive changes in children and adults with moyamoya disease

**DOI:** 10.1186/s12883-023-03520-z

**Published:** 2024-01-02

**Authors:** Min-Gyu Park, Jieun Roh, Sung-Ho Ahn, Jae Wook Cho, Kyung-Pil Park, Seung Kug Baik

**Affiliations:** 1 Department of Neurology, Pusan National University Yangsan Hospital, Research Institute for Convergence of Biomedical Science and Technology, Pusan National University School of Medicine, 20 Geumo-ro, Yangsan, 50612 Republic of Korea; 2 Department of Radiology, Pusan National University Yangsan Hospital, Pusan National University School of Medicine, Yangsan, Republic of Korea

**Keywords:** Moyamoya disease, Perivascular space, Glymphatic system, Cerebrovascular disorder

## Abstract

**Background:**

Dilated perivascular spaces (DPVS), known as one of imaging markers in cerebral small vessel disease, may be found in patients with moyamoya disease (MMD). However, little is known about DPVS in MMD. The purpose of this study was to investigate the distribution pattern of dPVS in children and adults with MMD and determine whether it is related to steno-occlusive changes of MMD.

**Methods:**

DPVS was scored in basal ganglia (BG) and white matter (WM) on T2-weighted imaging, using a validated 4-point semi-quantitative score. The degree of dPVS was classified as high (score > 2) or low (score ≤ 2) grade. The steno-occlusive changes on MR angiography (MRA) was scored using a validated MRA grading. Asymmetry of DPVS and MRA grading was defined as a difference of 1 grade or higher between hemispheres.

**Results:**

Fifty-one patients with MMD (mean age 24.9 ± 21.1 years) were included. Forty-five (88.2%) patients had high WM-DPVS grade (degree 3 or 4). BG-DPVS was found in 72.5% of all patients and all were low grade (degree 1 or 2). The distribution patterns of DPVS degree in BG (*P* = 1.000) and WM (*P* = 0.767) were not different between child and adult groups. The asymmetry of WM-DPVS (26%) and MRA grade (42%) were significantly correlated to each other (Kendall’s tau-b = 0.604, *P* < 0.001).

**Conclusions:**

DPVS of high grade in MMD is predominantly found in WM, which was not different between children and adults. The correlation between asymmetry of WM-DPVS degree and MRA grade suggests that weak cerebral artery pulsation due to steno-occlusive changes may affect WM-DPVS in MMD.

## Introduction

Moyamoya disease (MMD) is a chronic cerebrovascular disease characterized by progressive stenosis at the terminal portion of the internal carotid artery and abnormal vascular network at the base of the brain. During the course of MMD, transient ischemic attack, cerebral infarction, and cerebral hemorrhage are common, but there is no definitive therapy available to stop or slow the progression of MMD [1]. Although the most prominent change in MMD occurs in proximal intracranial arteries, inconspicuous changes may occur in spaces around small cerebral vessels called perivascular spaces (PVS) [2]. A few studies reported that dilated PVS (DPVS) can be found in patients with MMD [3, 4]. However, the mechanism and clinical significance of DPVS in MMD have not been well evaluated.

PVS is a normal physiological space filled with interstitial fluid around perforating cerebral vessels [5, 6]. DPVS is found in 1.6–3% of healthy individuals, so it had been considered a benign finding [7, 8]. However, accumulating evidences have suggested that DPVS is associated with cerebral small vessel disease (CSVD) and is currently considered as one of the neuroimaging markers of CSVD [9]. Recently, beyond the role of the neuroimaging marker of CSVD, it has been suggested that DPVS may contribute to cognitive impairment in CSVD and Alzheimer’s disease (AD) by disruption of perivascular drainage of amyloid β [6]. A recent study demonstrated a higher incidence of DPVS was in patients with intra- or extracranial atherosclerosis [10]. It was suggested that such a result may be related to decreased cerebral blood flow due to cerebral atherosclerosis. Both MMD and cerebral atherosclerosis cause narrowing of major cerebral arteries. Therefore, if decreased cerebral blood flow is related to the development of DPVS in cerebral atherosclerosis, the development of DPVS in MMD may be related to it [10]. So, we hypothesized that the incidence of DPVS would increases in children and adults with MMD as the degree of stenosis of major cerebral arteries increases.

## Methods

### Patients

Patients diagnosed with MMD at Pusan National University Yangsan Hospital from 2008 to 2018 were considered as subjects for this study. The diagnosis of MMD was based on the findings of magnetic resonance imaging (MRI), MR angiography (MRA), and/or digital subtraction angiography. We excluded the following patients: (1) patients with 1.5 T MRI; (2) patients whose DPVS could not be identified on MRI due to extensive parenchymal damage from previous cerebral infarction or hemorrhage; (3) patients without MRI or MRA before bypass surgery; (4) patients with other causes of arterial occlusion, such as arteriosclerosis, cerebral vasculitis, or intracranial artery dissection; (5) patients with moyamoya-like vasculopathy in the setting of another syndromic condition as neurofibromatosis type 1, tuberous sclerosis, or Down, Turner and Noonan syndromes. The selected MMD patients were divide into a child group (age < 16) and an adult group (age ≥ 16). Baseline clinical data, including age, sex, conventional vascular risk factors, and clinical manifestations were collected for all patients. This study was approved by the Ethics Committee of Pusan National University Yangsan Hospital (Institutional Review Board number: 05-2017-147).

### MRI acquisition

Patients were imaged with 3 T clinical MRI systems (Verio or Skyra; Siemens Healthineers, Germany). T2-weighted image parameters were as follows: repetition time = 4,650 milliseconds; echo time = 85 milliseconds; matrix number = 176 × 176; field of view = 190 × 220; flip angle = 120°; slice thickness = 5 mm; and intersection gap = 2 mm. Time-of-flight MRA was performed using a three-dimensional sequence with the following parameters: echo time = 3.7 ms, repetition time = 21 ms, flip angle = 18°, field of view = 167 × 260 mm, slice thickness = 0.5 mm, and space between slices = 20 mm.

### Imaging analysis

DPVS was defined as a round, ovoid, or linear structure (a maximum diameter < 2 mm) with a smooth boundary, showing cerebrospinal fluid-like signals on T2-weighted image, and located in areas supplied by perforating arteries [11]. In order to exclude the possibility of lacunes, those with a typical vascular shape and following the orientation of perforating vessels were regarded as DPVS [7]. For DPVS in the BG (BG-DPVS), DPVS 1–4 was scored as degree 1, DPVS 5–9 was degree 2, DPVS 10–19 was 3, and DPVS of 20 or more was rated as degree 4 [12]. For DPVS in the WM (WM-DPVS), DPVS 1–9 was rated as degree 1, DPVS 10–19 was degree 2, DPVS 20–39 was 3, and DPVS of 40 or more was rated as degree 4 [12]. We divided degrees of DPVS into low (degree 1 and 2) and high (degree 3 and 4) grade. When the DPVS degree on the right and left sides differed by more than one degree, they were classified as asymmetric, and the high degree was set as the final degree.

We used the MRA scoring system presented by Houkin et al. without modification to evaluate the severity of steno-occlusive lesion [13]. MRA scores for each hemisphere were assessed in 4 main intracranial arteries as follow: (1) internal cerebral artery (ICA); 0 = normal or minimum equivocal change of the intracranial ICA, 1 = apparent stenosis at the intracranial ICA, 2 = discontinuity of the signal of the C1 portion, 3 = no depiction of the intracranial ICA; (2) middle cerebral artery (MCA); 0 = normal or minimum equivocal change of the horizontal portion of the MCA, 1 = stenosis of the horizontal portion of the MCA with normal or equivocal signal diminishment of its distal branches, 2 = discontinuity of signal of the horizontal portion of the MCA and decrease of the signal of distal MCA, 3 = no depiction of most of the MCA territory; (3) anterior cerebral artery (ACA); 0 = normal signal intensity of the A2 and its distal branches, 1 = signal decrease or loss of the A2 and its distal branches, 2 = no depiction of the ACA; (4) posterior cerebral artery (PCA); 0 = normal or equivocal stenotic change of the P2 and its distal branches, 1 = signal decrease or loss of P2 and its distal branches diminishes, 2 = no depiction of the PCA. Therefore, in each cerebral hemisphere, the minimum MRA score is 0 and the highest MRA score is 10. The MRA score was classified into four grades as follow: grade 1, MRA score of 0–1; grade 2, MRA score of 2–4; grade 3, MRA score 5–7; grade 4, MRA score 8–10 [13]. When the MRA grades between right and left sides differed by more than 1 grade, they were classified as an asymmetry, and the higher grade was determined as a final grade.

**Fig. 1:**
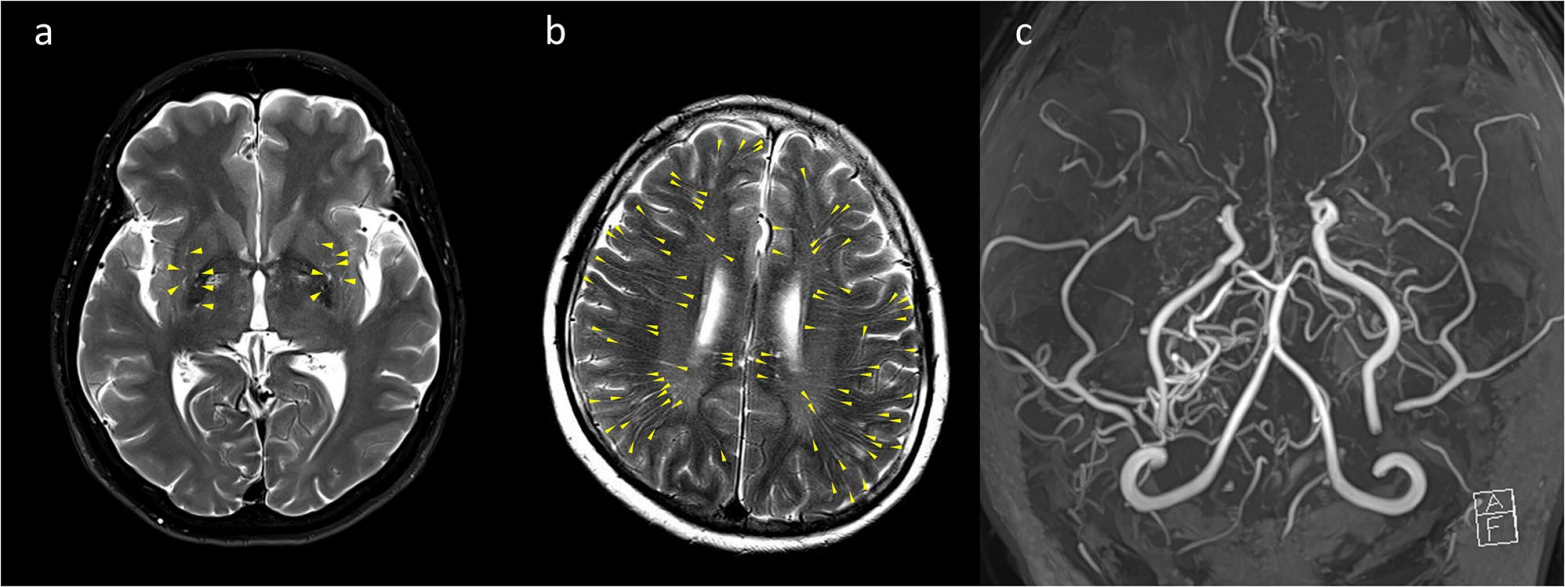
Representative example of dilated perivascular spaces (DPVS) and MRA grade in 52-year-old female with hypertension, diabetes, and hyperlipidemia. (**a**) Less than 10 DPVS (arrowheads, degree of 2) are observed in both basal ganglia. (**b**) More than 40 DPVS (arrowheads, degree of 4) are observed on both white matters: it can be defined as symmetry between the DPVS degrees of both sides. (**c**) The right side have the MRA score of (ICA, 1 + MCA, 3 + ACA, 2 + PCA, 0) and the MRA grade of 3. The left side have the MRA score of 7 (ICA, 2 + MCA, 3 + ACA, 2 + PCA, 0) and the MRA grade of 3. Therefore, it can be defined that both MRA grades are symmetric with each other

**Fig. 2:**
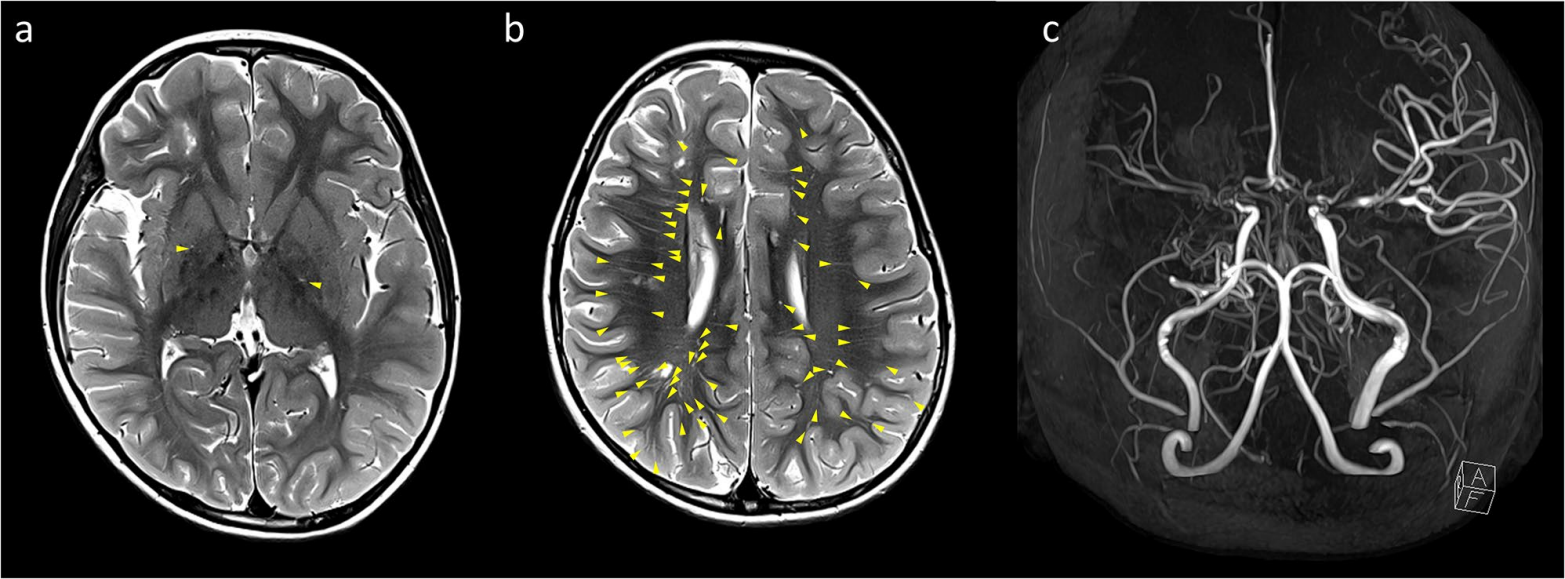
Representative example of dilated perivascular spaces (DPVS) and MRA grade in 5-year-old male without any vascular risk factors. (**a**) Less than 5 DPVS (arrowheads, degree of 1) are observed in both basal ganglia. (**b**) More than 40 DPVS (arrowheads, degree of 4) are observed in the right white matter, and less than 40 DPVS (arrowheads, degree of 3) is observed in the left white matter: it can be defined as asymmetry between the DPVS degrees of both sides. (**c**) The right side have the MRA score of 5 (ICA, 2 + MCA, 2 + ACA, 1 + PCA, 0) and the MRA grade of 3. The left side have the MRA score of 4 (ICA, 1 + MCA, 1 + ACA, 1 + PCA, 0) and the MRA grade of 2. Therefore, it can be defined that both MRA grades are asymmetric with each other

The DPVS degree were independently classified by stroke neurologist with 11 years of experience and neuroradiologist with 8 years of experience who were blinded to each other’s findings. The MRA score were independently classified by two stroke neurologists, each with 20 years and 9 years of experience in stroke neurology, who were blinded to each other’s findings. The discordance between grades was decided by consultation with a neuroradiologist with 26 years of experience. Representative cases of DPVS and MRA score are shown in Figs. 1 and 2.

### Statistical analysis

We performed appropriate univariate tests to compare clinical and radiological data between child and adult groups, and between patients with high and low degrees of DPVS. Pearson’s chi-square test or Fisher’s exact test were used for categorical variables, and Mann-Whitney U test for continuous variables. The interobserver and intraobserver variabilities of DPVS degree and the interobserver variability of MRA score were analyzed by the kappa statistic. Kendall rank correlation coefficient was used to measure the ordinal association between DPVS degree and MRA grade asymmetry. We analyzed the data using Statistical Package for Social Sciences software for windows (Version 26; IBM, Armonk, New York). Statistical significance was defined as *P* < 0.05.

## Results

After excluding a total of 34 patients (25 patients with 1.5T MRI, 4 patients with previous extensive parenchymal damage, 5 patients without preoperative MRI), 51 patients with MMD were finally selected in the study. Clinical characteristics and demographic features of included patients are summarized in Table 1. The patients included 13 men (25.5%) and 38 women (74.5%) with ages ranging from 1.6 to 66 years. Twenty-five patients (49.0%) were under 16 years of age. There was female predominance (92.4%, *P* = 0.004) in the adult group. In the child group, no patient had conventional vascular risk factors. There were no significant differences of clinical manifestations between the child and adult groups. Cerebral infarction (11 patients in the child group and 13 patients in the adult group) was the most common frequent, followed by transient ischemic attack (9 patients in the child group and 7 patients in the adult group), headaches (4 patients in the child group and 3 patients in the adult group), intracerebral hemorrhage (1 patient each in the both groups), dizziness (1 patient in the adult group), and syncope (1 patient in the child group).

**Table 1:** The clinical and demographic characteristics of the subject

	Total (n = 51)	Child (n = 26)	Adult (n = 25)	*P* value
Age, years (mean ± SD)	24.9 ± 21.1	7.0 ± 3.6	42.0 ± 15.9	< 0.001*
Female, n (%)	38 (74.5)	14 (56)	24 (92.3)	0.004*
Hypertension, n (%)	8 (15.7)	0	8 (30.8)	0.004*
Diabetes, n (%)	6 (11.8)	0	6 (23.1)	0.023
Hyperlipidemia, n (%)	4 (7.8)	0	4 (15.4)	0.110
Smoking, n (%)	2 (3.9)	0	2 (7.7)	0.490
**Clinical manifestations**				
Cerebral infarction, n (%)	24 (47.1)	11 (44)	13 (50)	0.886
TIA, n (%)	16 (31.4)	9 (36)	7 (27.0)	0.611
ICH, n (%)	2 (4.0)	1 (4)	1 (4.0)	1.0
Others (headache, dizziness, or syncope), n (%)	9 (17.7)	5 (20)	4 (15.4)	0.465

*SD*, standard deviation; *TIA*, transient ischemic attack; *ICH*, intracerebral hemorrhage

*P < 0.05

Imaging characteristics of included patients are summarized in Table 2. Interpretation of WM-DPVS (k = 0.82, P < 0.001), BG-DPVS (k = 0.88, *P* < 0.001), and MRA score (k = 0.96, *P* < 0.001) showed good agreement between the observers. Interpretation of DPVS for each observer showed also good agreement: WM-DPVS (k = 0.90, P < 0.01) and BG-DPVS (k = 0.94, *P* < 0.01) in observer 1; WM-DPVS (k = 0.90, P < 0.01) and BG-DPVS (k = 0.93, *P* < 0.01) in observer 2. 88.2% (44 patients) of all patients had high grade (degree 3 and 4) of WM-DPVS. BG-DPVS was found in 72.5% of all patients and all were low grade (degree 1 or 2) of BG-DPVS. There was no difference in the distribution pattern of the degree of WM-DPVS (*P* = 0.767) and BG-DPVS (*P* = 0.100) between child and adult groups. The median of MRA score was 5 (range, 2–9) in the child group and 6 (range, 3–9) in the adult group. The MRA score tended to be higher in the adult group (*P* = 0.033), but the MRA grades were not different between child and adult groups (*P* = 0.050).

**Table 2:** The imaging characteristics of the subject

		Total (n = 51)	Child (n = 26)	Adult (n = 25)	*P* value
WM-dPVS, n (%)					0.767
Low grade	Degree 1, 1–9	2 (3.9)	1 (4)	1 (3.8)	
	Degree 2, 10–19	4 (7.8)	2 (8)	2 (7.7)	
High grade	Degree 3, 20–39	20 (39.2)	8 (32)	12 (46.2)	
	Degree 4, ≥ 40	25 (49.0)	14 (56)	11 (42.3)	
BG-dPVS, n (%)					1.000
Low grade	Degree 1, 1–4	45 (88.2)	22 (88)	23 (88.5)	
	Degree 2, 5–9	6 (11.8)	3 (12)	3 (11.5)	
High grade	Degree 3, 10–19	0	0	0	
	Degree 4, ≥ 20	0	0	0	
MRA score, median (range)		6 (2–9)	5 (2–9)	6 (3–9)	0.033
MRA grade, median (range)		3 (2–4)	3 (2–4)	3 (2–4)	0.050

*WM-dPVS*, dilated perivascular spaces in the white matter; *BG-dPVS.* dilated perivascular spaces in the basal ganglia; *MRA*, MR angiography

Comparison of characteristics between high and low WM-DPVS grades is shown in the Table 3. There was no association between high degree of WM-DPVS and vascular risk factors such as age (*P =* 1.000), hypertension (*P =* 1.000), diabetes (*P* = 0.548), hyperlipidemia (*P* = 0.404), and smoking (*P =* 1.000). There was no difference in MRA score (*P* = 0.180) and MRA degree (*P* = 0.086) between low and high WM-DPVS grades. Asymmetry of WM-DPVS degree was confirmed in 26% of all patients, and asymmetry of BG-DPVS was not found in all patients. Asymmetry of MRA grade was showed in 42% of all patients. In all patients with asymmetry of WM-DPVS degree, the side of higher WM-DPVS degree was ipsilateral to the side of higher MRA grade. There was a significant positive correlation between the asymmetry of WM-DPVS degree and MRA grade (Kendall’s tau-b = 0.604, *P* < 0.001).

**Table 3:** The comparison between low and high WM-dPVS grades

	WM-dPVS, low (n = 6)	WM-dPVS, high (n = 45)	*P* value
Child, n (%)	3 (50)	22 (48.9)	1.000
Female, n (%)	6 (100)	32 (71.1)	0.318
Hypertension, n (%)	1 (16.7)	7 (15.6)	1.000
Diabetes, n (%)	1 (16.7)	5 (11.1)	0.548
Hyperlipidemia, n (%)	1 (16.7)	3 (6.7)	0.404
Smoking, n (%)	1 (16.7)	2 (4.4)	1.000
MRA score, median (range)	5 (4–7)	6 (2–9)	0.180
MRA grade, median (range)	3 (2–3)	3 (2–4)	0.086

*WM-dPVS*, dilated perivascular spaces in the white matter; *MRA*, MR angiography

## Discussion

In this study, WM-DPVS of high grade was observed in 88.2% of all patients, and there was no difference in the incidence of WM-DPVS between children and adults with MMD. Medullary arteries pass through PVS in WM [14]. Previous several studies have suggested that periventricular anastomosis develops between perforating arteries and medullary arteries in MMD [15, 16]. It is possible that change of blood flow through medullary arteries might cause dilatation of PVS of WM in MMD. Periventricular anastomosis might be a result of response to long-standing ischemia, which might mean that medullary arteries have been exposed to long-standing hemodynamic stress [15]. Therefore, WM-DPVS in MMD might represent the long-lasting hemodynamic stress of medullary arteries.

Recently published studies raised the possibility that DPVS in MMD may be associated with weak cerebral arterial pulsation [3, 4]. PVS is a key anatomical structure that constitutes the glymphatic system, and it plays an important role in drainage of interstitial fluid and solutes in the central nervous system [5]. Arterial pulsation is known to provide driving force to drain the interstitial fluid and solutes through PVS [5, 17]. Factors such as aging and hypertension weaken cerebral arterial pulsation, which has been pointed out as one of the reasons why amyloid β is not removed from AD brain [18, 19]. In MMD, arterial pulsation may be attenuated due to the steno-occlusive changes of the proximal intracranial arteries, which may result in suppression of interstitial fluid drainage through PVS and widening of PVS [3, 4]. In this study, when the severity of steno-occlusive changes on MRA were different between left and right sides, more WM-DPVS was found in the hemisphere with a higher MRA grade. More severe steno-occlusive changes could further weaken arterial pulsation, which might have more impact on DPVS [20]. Therefore, such result might be indirect evidence that weak arterial pulsation is associated with DPVS in MMD. However, while asymmetry in MRA grade is found in 42% of patients, asymmetry in DVPS is present only in 26% of all cases. It is not easy to reveal the reason why there is such a discrepancy between asymmetries in MRA grade and DVPS. MMD is a slowly progressive disease. It usually begins on both sides, but may not begin at the same time and may not deteriorate at the same speed [21–23]. Even if the causal relationship between steno-occlusive change and DPVS is not understood fully in MMD, it may require a considerable time for steno-occlusive change to expand the PVS. Therefore, it is assumed that each of such different time intervals might be associated with discrepancy between asymmetries in MRA grade and DVPS in this study.

Blood-brain barrier (BBB) dysfunction may be also associated with DPVS. BBB is one of the important anatomical structures that form PVS, which plays a complementary role with the glymphatic system in clearing waste from the brain [24]. Recent studies have shown that BBB leakage increases in AD and CSVD [25, 26]. Increased BBB leakage contributes to stagnation of the interstitial fluid, which could lead to DPVS [27]. The DPVS found in brain with AD and CSVD might be the result of such mechanism [25–27]. A recent study showed in vivo BBB damage by visualizing the extravasation of sodium fluorescein into the cortical parenchyma during bypass surgery in MMD patients [28]. Therefore, dysfunction of BBB may be related to DPVS in MMD, and further research is needed to confirm this.

In this study, it is difficult to clarify why more DPVS is observed in WM than in BG. There are two possible hypotheses for this finding. First, it is the histological differences between PVS in BG and WM [5, 7, 29]. In WM, PVS is formed between one layer of leptomeninges and arterial wall. In BG, PVS is formed between the two layers of leptomeninges. Due to such histological differences, the dynamics of interstitial fluid through PVS in BG may be different from that in WM [30]. Although there is no direct evidence to support this hypothesis, a recently published MRI study showed remarkable indirect evidence. The study revealed the difference of signal intensity on pre-contrast images and enhancement on post-contrast images between PVS in BG and WM [30]. The authors of the study pointed out that the histological differences between PVS in BG and WM may be responsible for such findings. The second hypothesis is that the distance from the steno-occlusive lesions of MMD to PVS affects the distribution of DPVS. The steno-occlusive changes of MMD could affect pulsation of both lenticulostriate arteries and medullary arteries. The medullary arteries are farther from the steno-occlusive lesions of MMD than the lenticulostriate arteries. Therefore, the long distance from steno-occlusive lesions of MMD may weaken pulsation of the medullary arteries than that of the lenticulostriate arteries [31].

In this study, children with MMD have DPVS distribution of the same pattern as adults with MMD. Children with MMD have no conventional vascular risk factors, especially hypertension. Hypertension is known to be related to DPVS in adults, especially DPVS in BG [32, 33]. Moreover, in this study, DPVS in both children and adults with MMD were observed to be more prevalent in WM than in BG. Therefore, the pathogenesis of DPVS in MMD may be different from that of DPVS in adults with hypertension. Of course, it is not yet clear why the distribution pattern of DPVS in MMD is different from adults with hypertension. Answers to such questions are needed for clinicians to understand the role of DPVS in various neurological diseases such as MMD, CVSD, and AD.

Our study has some limitations. First, the number of patients with MMD included in this study is not large. This is one of the common problems in studies on rare diseases. So, collaborative studies involving several institutions will be needed for a deep investigation on DPVS in MMD. Second, due to the characteristics of the tertiary medical institution, the number of early-stage MMD patients included in this study is not large. Examining the development of DPVS from early- to late-stage MMD will be meaningful to find out the clinical significance and the pathogenesis of DPVS in MMD. Finally, in our study, visual rating scales were used to measure the burden of DPVS. A methodology to measure the burden of DPVS has not yet been precisely defined. Recently, a study that attempted quantitative analysis of DPVS has been published, and we think that such a method may be useful for subsequent studies on DPVS [34].

## Conclusions

In moyamoya disease, dilated perivascular spaces of high grade are predominantly found in white matter, which was not different between children and adults. The correlation between asymmetry of dilated perivascular spaces in white matter and MRA grade asymmetry suggests that weak artery pulsation due to steno-occlusive changes may affect dilated perivascular spaces. Further studies are needed to clarify the clinical significance of dilated perivascular spaces in moyamoya disease.

## Data Availability

The data sets used and/or analyzed during the current study are available from the corresponding author on reasonable request.

## Abbreviations

MMD: moyamoya disease
PVS: perivascular spaces
DPVS: dilated perivascular spaces
BG: basal ganglia
WM: white matter
MRI: magnetic resonance imaging
MRA: magnetic resonance angiography
CSVD: cerebral small vessel disease
AD: Alzheimer’s disease
ICA: internal carotid artery
MCA: middle cerebral artery
ACA: anterior cerebral artery
PCA: posterior cerebral artery
